# Prenatal and Postnatal Cigarette Smoke Exposure Is Associated With Increased Risk of Exacerbated Allergic Airway Immune Responses: A Preclinical Mouse Model

**DOI:** 10.3389/fimmu.2021.797376

**Published:** 2021-12-23

**Authors:** Hamed Janbazacyabar, Jeroen van Bergenhenegouwen, Johan Garssen, Thea Leusink-Muis, Ingrid van Ark, Marthe T. van Daal, Gert Folkerts, Saskia Braber

**Affiliations:** ^1^ Division of Pharmacology, Utrecht Institute for Pharmaceutical Sciences, Faculty of Science, Utrecht University, Utrecht, Netherlands; ^2^ Global Center of Excellence Immunology, Danone Nutricia Research, Utrecht, Netherlands

**Keywords:** pregnancy, cigarette smoke, immune response, house dust mite, allergy, airway

## Abstract

Increased exposure to household air pollution and ambient air pollution has become one of the world’s major environmental health threats. In developing and developed countries, environmental cigarette smoke (CS) exposure is one of the main sources of household air pollution (HAP). Moreover, results from different epidemiological and experimental studies indicate that there is a strong association between HAP, specifically CS exposure, and the development of allergic diseases that often persists into later life. Here, we investigated the impact of prenatal and postnatal CS exposure on offspring susceptibility to the development of allergic airway responses by using a preclinical mouse model. Pregnant BALB/c mice were exposed to either CS or air during pregnancy and lactation and in order to induce allergic asthma the offspring were sensitized and challenged with house dust mite (HDM). Decreased lung function parameters, like dynamic compliance and pleural pressure, were observed in PBS-treated offspring born to CS-exposed mothers compared to offspring from air-exposed mothers. Maternal CS exposure significantly increased the HDM-induced airway eosinophilia and neutrophilia in the offspring. Prenatal and postnatal CS exposure increased the frequency of Th2 cells in the lungs of HDM-treated offspring compared to offspring born to air-exposed mothers. Offspring born to CS-exposed mothers showed increased levels of IL-4, IL-5 and IL-13 in bronchoalveolar lavage fluid compared to offspring from air-exposed mothers. Ex-vivo restimulation of lung cells isolated from HDM-treated offspring born to CS-exposed mothers also resulted in increased IL-4 production. Finally, serum immunoglobulins levels of HDM-specific IgE and HDM-specific IgG1 were significantly increased upon a HDM challenge in offspring born to CS-exposed mothers compared to offspring from air-exposed mothers. In summary, our results reveal a biological plausibility for the epidemiological studies indicating that prenatal and postnatal CS exposure increases the susceptibility of offspring to allergic immune responses.

## Introduction

According to the World health Organization (WHO), approximately 300 million people currently suffer from asthma (1), and it is estimated that the global prevalence of asthma increases by 50% every decade with the most striking increase among children (2, 3). Multiple factors have been shown to be involved in increased incidence of allergic asthma, including genetic factors. However, the rapid rise in asthma and allergy incidence throughout the world implies that, in addition to hereditary factors, environmental factors, including air pollution, may also play a role (4, 5). Allergic asthma is the most common form of pediatric chronic pulmonary inflammatory diseases in children characterized by sensitization to specific allergens, airway eosinophilia, high level of immunoglobulin (Ig) E and complex immune responses in the lung starting with allergen specific T helper responses to non-infections antigens (e.g. house dust mite) (6). Although several factors participate in the development of asthma, emerging evidence from several epidemiological and experimental studies indicate that maternal exposure to environmental factors, such as air pollution and cigarette smoke (CS) exposure may contribute to the development of childhood asthma (7–13). Despite the well documented adverse health outcomes of maternal CS exposure, 20-30% of women who smoke, continue smoking during pregnancy and lactation (14, 15). Besides active smoking, passive smoking, also known as environmental tobacco smoke exposure (ETS), is a major contributor to household air pollution (HAP) (16). Several lines of research have demonstrated that ETS, a main public health concern, leads to development of several diseases including asthma and cancers (17–19). Particularly, prenatal ETS exposure has been strongly linked to allergic asthma, respiratory dysfunction and infection in children (20, 21). Although there is a strong association between maternal smoke exposure and adverse birth outcomes, the mechanism by which maternal CS could lead to the development of several diseases later in life still remains unclear. However, it becomes increasingly evident that adverse effects during the first 1000 days (from conception until the first two year of life) may have long-life health consequences (22, 23). Studies have indicated that the developing fetus and children are more vulnerable to environmental exposure due to their immature detoxification pathways and undeveloped immune system (24–26). Therefore, early life exposure to a variety of environmental factors, including air pollution and CS exposure, can predispose neonates to diseases and affect the dynamics of immune responses in later life (27, 28). CS and air pollution in general are an extremely complex and dynamic mixture of substances containing both gasses and particulate matter (PM). Numerous studies have indicated that fine/ultrafine PM can cross epithelial barriers, including the placental barrier, suggesting that exposure to environmental contaminants might already take place in the womb (29–31).

Epidemiological studies link CS exposure during the first 1000 days to increased adverse respiratory health effects, such as increased incidence of lower respiratory tract illness, airway hyper responsiveness, impaired lung function and the development of asthma and bronchitis in later life (32, 33). A clinical study showed that prenatal CS exposure during the third semester of pregnancy is associated with increased risk of asthma development and exacerbation as observed by reduced lung function parameters (34).

However, in depth studies are needed to fully elucidate the impact of prenatal and postnatal CS exposure on the onset of asthma and corresponding immune responses later in life. Therefore, the present study aimed to further investigate the impact of prenatal and postnatal CS exposure on offspring susceptibility to the development of allergic airway responses by utilizing a house dust mite (HDM)-induced murine asthma model. HDM is one of the most common environmental aeroallergens and this model demonstrates many characteristics of human allergic asthma, including the presence of eosinophilic airway inflammation and a Th2-type allergic inflammatory response (35, 36). To this end, we compared the effect of HDM treatment on inflammatory cell influx into bronchioalveolar lavage, pulmonary Th1 and Th2 cell frequency, airway cytokines and lung function parameters in offspring prenatally and postnatally exposed to CS and their air-exposed littermates. In addition, HDM specific humoral immune responses, including HDM-IgE and HDM-IgG1, were measured.

## Materials and Methods

### Animals

Sixty female and thirty male BALB/c by JIco mice at 8 weeks of age were purchased from Charles River Laboratories (Someren, the Netherlands). Upon arrival, mice were housed in groups (Female: 6/cage; Male: 5/cage used for mating) in filter-topped makrolon cages (22 cm×16 cm×14 cm, floor area 350 cm2, Tecnilab- BMI, Someren, the Netherlands) with wood-chip bedding (Tecnilab- BMI, Someren in the Netherlands) and tissues (VWR, the Netherlands) were available as cage enrichment at the animal facility of Utrecht University. Mice were kept under a light/dark cycle of 12 h/12 h (lights on from 7.00 am–7.00 pm) at controlled relative humidity (relative humidity of 50–55%) and temperature (21 ± 2°C) with ad libitum access to tap water and pelleted food (AIN-93G, Ssniff Spezialdiäten, Soest, Germany). After mating, cardboard houses were added to the cages. More details are provided in the sections below.

This experiment was performed in accordance with institutional guidelines of Utrecht University and all animal procedures related to the purpose of the research were approved by the local Animal Welfare Body under license of the national competent authority (‘*Centrale Commissie Dierproeven’, CCD*), securing full compliance the European Directive 2010/63/EU for the use of animals for scientific purposes.

### Mating, Gestation, and Smoke-Exposure

**Figure 1:**
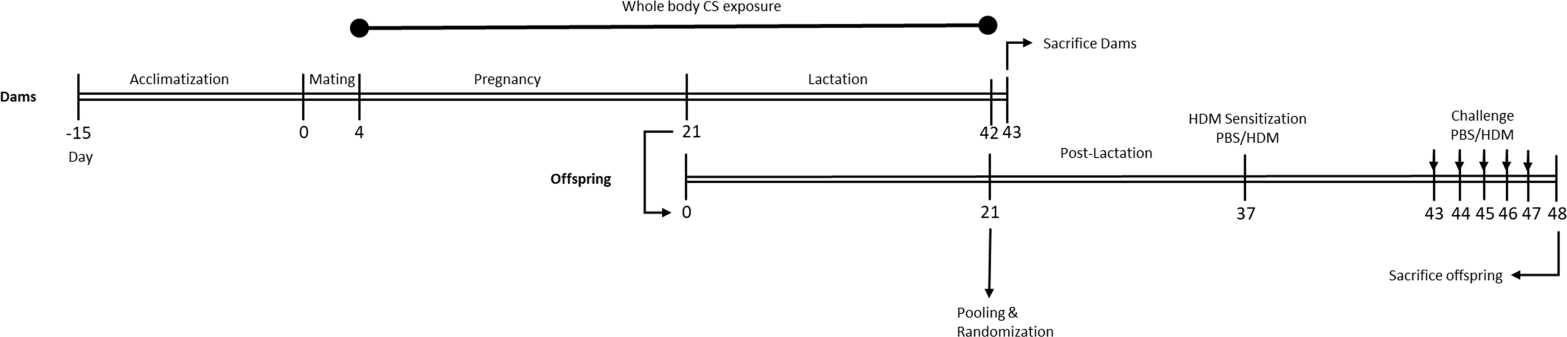
Experimental timeline. Prenatally and postnatally air- and CS-exposed offspring were intranasally (i.n.) sensitized with PBS or house dust mite (HDM) on day 37 and challenged on days 43-47 intranasally with PBS or HDM.

Following the acclimatization period (two weeks) and one day prior to mating, female mice were randomly housed two per cage (from the same cohort) and allocated to the control (n=30) or CS exposure group (n=30). The next day, a single male mouse was introduced to two females (considered as pregnancy day [P] 0). After 4 days of mating, male mice were removed from the female cages. Afterwards, from pregnancy day 4 (P4) until the end of lactation, the female mice were placed daily in whole-body chambers (8 animals/chamber, pups remained in the cage with extra tissues to maintain temperature) and exposed to either air or diluted mainstream CS from the reference cigarettes 1R6F (University of Kentucky, Lexington, Kentucky) using smoke apparatus as described previously (37). Exposures were conducted for 45 min/day (resembling 14 cigarettes/day), 7 days/week, for 6 weeks (from P4 until the end of lactation) (38). Carbon monoxide (CO) levels was measured and monitored continuously and was approximately 300-400 ppm. The mass concentration of cigarette smoke total particulate matter (TPM) was determined by gravimetric analysis using a type A/E glass fiber filter (PALL life sciences, Mexico). Briefly, the glass filter was weighed and placed in the filter holder. Next, CS was passed through the filter. TPM was calculated by subtracting the basal filter weight from the CS-exposed filter divided by the volume of the filter holder and expressed as μg/L (39). The TPM concentration in the smoke exposure box generated by 14 cigarettes reached approximately 828 μg/L (828 ± 4.5 μg/L) as previously described (37). At birth, the two mothers with their pups were maintained in filtered-top cages, and the numbers of viable offspring, along with the approximate duration of gestation, were determined (the litter size for air- and smoke-exposed groups was 4.66 and 4.07, respectively) (37). After 3 weeks of lactation, pups were weaned and sexed. After weaning, prenatally and postnatally air and CS-exposed pups (male and female) were separately (air and smoke) pooled and randomly assigned to the prenatal air or CS exposure groups and part of the pups were sacrificed right after weaning as described in our previously published paper (37), and the rest of the pups were used for the current study and assigned to HDM or PBS treatment groups. Moreover, offspring body weight was measured for three consecutive weeks (once per week) and a reduced body weight in PBS and HDM-treated offspring born to CS-exposed mothers compared to offspring from air-exposed mothers (**Supplementary Figure 1**) was observed. An overview of the experimental design is presented in **Figure 1**.

### HDM Model

Two weeks after weaning, mice were intranasally sensitized with PBS in presence or absence of 1 μg HDM (Greer Laboratories, Lenoir, USA) on day 37 and challenged with PBS or 10 μg HDM on days 43 to 48 under light isoflurane anesthesia (**Figure 1**) (40).

### Airway Responsiveness Measurement

One day after the final HDM challenge, mice were anesthetized by an intraperitoneal injection with a mixture of ketamine (Vetoquinol S.A., France; 125 mg/kg) and medetomidine (Pfizer, The Netherlands; 0.4 mg/kg). EMKA invasive measurement of dynamic resistance (EMKA Technologies, Paris, France) in response to increasing doses of methacholine (acetyl-β-methyl-choline chloride, Sigma-Aldrich) (0–25 mg/mL, 10% puff for 10 s) was used to determine lung function (35). This is in line with a previous murine house dust mite-induced asthma model where lung function (lung resistance) was measured 24h after the last HDM challenge (37). Data are expressed as average lung resistance (R_L_), dynamic compliance (Cdyn) and pleural pressure (dPpl) in cm H2O/mL*sec-1 and the area under the curve (AUC) as the indexes (40).

### Blood Sampling and Serum Preparation

Immediately after measuring the lung function under anesthesia, mice were sacrificed using an overdose of pentobarbital (200 mg/kg; NembutalTM, Ceva Santé Animale, Naaldwijk) by intraperitoneal injection and blood samples were directly taken *via* cardiac puncture. Afterwards, the blood samples were coagulated for 30 min at room temperature, centrifuged at 10,000 rpm for 10 min and the serum samples were stored at -20°C until further analysis.

### Bronchoalveolar Lavage

After lung function measurement and immediately following euthanasia, a cannula was placed into the trachea and lungs were gently lavaged four times with pre-warmed pyrogen-free saline (0.9% NaCl, 37°C) in situ. Lungs were first lavaged with pyrogen-free saline (0.9%NaCl, 37°C) supplemented with a protease inhibitor cocktail tablet (Complete Mini, Roche Diagnostics, Mannheim, Germany). To maximize the cell harvesting from the bronchioloalveolar lavage fluid (BALF), lungs were additionally lavaged for three more times with saline solution (0.9% NaCl, 37°C). The BALF was cooled on ice and centrifuged at 4°C (400 × g, 5 min). The supernatant of the first lavage was used for cytokine measurement and the cell pellets were pooled per animal, resuspended in 150 μl cold PBS and counted under light microscopy using a Bürker-Türk chamber (magnification 100×). Differential counts of BALF cells were performed on air-dried cytospin preparations stained with DiffQuik™ (Merz & Dade A.G., Düdingen, Switzerland) and numbers of total cell, macrophages, neutrophils, eosinophils, lymphocytes and granulocytes were identified according to standard morphology.

### *Ex Vivo* Lung Restimulation With HDM

Fresh lungs were collected aseptically, cut into small pieces and homogenized using enzymatic digestion buffer (DNase I and Collagenase A, Roche Diagnostics). After 30 min incubation, the enzymatic digestion process was stopped by adding fetal calf serum (FBS, Bodinco, The Netherlands). Lung pieces were gently passed through a 70 µm nylon cell strainer. The collected cell suspensions were washed and resuspended in RPMI 1640 medium (Lonza, The Netherlands) and supplemented with 10% heat-inactivated FBS and penicillin (100 U/mL)/streptomycin (100 µg/mL; Sigma-Aldrich). Total cell number was determined by using a Beckman Z1 coulter^®^ Particle Counter (Beckman, USA) and lung cells (10^6^ cells/ml) were cultured in 96-well U-bottom plates (Greiner Bio-One B. V., The Netherlands), either with medium or with medium supplemented with 50 μg/mL HDM (Greer Laboratories). After 4 days of culture at 37°C in 5% CO_2_, cell culture supernatant was collected and stored at −20°C until further analysis.

### Flow Cytometry

To determine T cell subsets in the lung, freshly isolated cells were blocked for 2 min at 4˚C using anti-mouse CD16/CD32 antibodies (Mouse BD Fc Block; BD Pharmingen, San Jose, CA, USA) and resuspended in FACS buffer (1% bovine serum albumin (BSA) in PBS). Subsequently, cells were washed and stained using combination of different antibodies against CD4-PerCP Cy5 (cat no. 45-0042, clone RM4-5), CD69-FITC (cat no. 11-0691, clone H1.2F3), GATA3-PE (cat no. 12-9966, clone TWAJ), Tbet-eFLUOR660 (cat no. 50-5825, clone eBio4D10), RORγt-PE (cat no. 12-6988, clone AFKJs-9), Foxp3-APC cat no: 17-5773, clone FJK-16s (eBioscience, San Diego, USA), CD8a-APC Cy7 (cat no. 557654, clone 53-6.7) and CD25-FITC (cat no. 553071, clone 7D4CD25 FITC) (BD, Breda, The Netherlands) for 60 min at room temperature. Viable cells were determined by a fixable viability Dye-eFluor^®^ 780 (eBioscience). For intracellular staining, cells were fixed and permeabilized using a fixation/permeabilization buffer set, according to manufacturer’s protocol (eBioscience). Flow cytometric acquisition was conducted with FACS Canto II (BD Biosciences) and results were analyzed using Flowlogic Software (Inivai Technologies, Victoria, Australia).

### Cytokines and Chemokines Measurement

IL-4, IL-5 and IL-13 concentrations were measured in cell culture supernatants and BALF samples by a ProcartaPlex**™** bead-based immunoassay (Life Technologies Europe BV) according to manufacturer’s protocol. The concentration of cytokines was expressed as picogram per milliliter.

### ELISA Serum HDM-Specific IgE and IgG1

To measure HDM-specific IgE and HDM-specific IgG1 in serum, specific ELISA plates (Corning 9018) were coated with HDM (50 mg/ml). After overnight incubation, the plates were washed and blocked with 1% w/v BSA in tween. Subsequently, plates were washed and incubated with diluted serum samples for 2h. Thereafter, plates were washed and incubated for 1.5h with 1 mg/ml biotin anti-mouse IgE (553419, BD Biosciences) or 1 mg/ml biotin anti-mouse IgG1 (553414, BD Biosciences). After washing, plates were incubated for 1h with streptavidin-HRP (Sanquin, Amsterdam). Plates were washed again and incubated with 3,3′5,5′‐tetramethylbenzidine (TMB) substrate solution (eBioscience) and after 20 min of reaction at room temperature and under dark conditions, this reaction was terminated by adding 2M H2SO4. The absorbance was measured using the Promega plate reader (Leiden, the Netherlands) at 450 nm.

### Statistical Analysis

Unless stated otherwise, results are expressed as mean ± standard error of mean (SEM). Data were statistically analyzed using a two-way ANOVA followed by a *post hoc* Bonferroni’s multiple comparisons test. A probability value P < 0.05 was considered significant. Statistical analyses were performed using GraphPad Prism software (version 6.04). The required sample size was calculated with G*Power v3.1.9 based on a power of 80% and α = 0,05 and primary outcome parameter derived from previous observations of lung resistance, which is the most relevant clinical parameter for HDM sensitization.

## Results

### Prenatal and Postnatal CS Exposure Affected Lung Function Parameters in the Offspring

**Figure 2:**
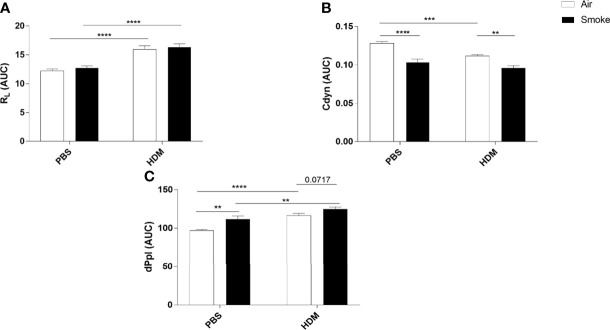
Prenatal and postnatal CS exposure affected lung function parameters in the offspring. Prenatally and postnatally air- and CS-exposed offspring were intranasally (i.n.) sensitized with PBS or house dust mite (HDM) on day 37 and challenged on days 43-47 intranasally with PBS or HDM and sacrificed one day after last HDM/PBS challenge. **(A)** Lung resistance (RL), **(B)** dynamic compliance (Cdyn) and **(C)** pleural pressure (dPpl) were measured after exposure to increasing doses of methacholine. Areas-Under-Curve (AUC) were calculated for statistical analysis as mean ± SEM. **P < 0.01, ***P < 0.001 and ****P < 0.0001; as analyzed with two-way ANOVA followed by Bonferroni’s multiple comparisons test, (n =10 mice/group).

Lung resistance (R_L_), lung dynamics (Cdyn) and pleural pressure (dPpl), as a measurement of lung function upon the HDM/PBS challenge, were determined in offspring prenatally and postnatally exposed to CS or air (**Figure 2**). As expected, HDM exposure increased the R_L_ in offspring prenatally and postnatally exposed to air or CS compared to PBS-treated mice (**Figure 2A**). However, no additional effect of HDM treatment on R_L_ values in offspring born to CS-exposed mothers compared to HDM-treated offspring born to air-exposed mothers was observed (**Figure 2B**).

To have a better understanding of the lung elasticity properties, Cdyn (known as pulmonary dynamic compliance) was measured (**Figure 2B**). HDM treatment significantly reduced Cdyn values in offspring born to air-exposed mothers. Prenatal and postnatal CS exposure induced a significant reduction in Cdyn values in PBS-treated offspring born to CS-exposed mothers compared to offspring from air-exposed mothers (**Figure 2B**). We did not observe any additional effect of HDM treatment on Cdyn values in offspring born to CS-exposed mothers compared to PBS-treated offspring born to CS-exposed mothers (**Figure 2B**).

HDM treatment alone significantly increased the dPpl value (**Figure 2C**). Offspring born to CS-exposed mothers showed a significant increase in dPpl after PBS treatment. Interestingly, HDM treatment caused a significant increase in dPpl in prenatally and postnatally CS-exposed offspring compared to PBS-treated offspring from CS-exposed mothers and compared to HDM-treated offspring from air-exposed mothers (p= 0.07) (**Figure 2C**).

### Prenatal and Postnatal CS Exposure Induced Additional Inflammatory Cell Infiltration Into the Lungs of Offspring Upon HDM Challenge

**Figure 3:**
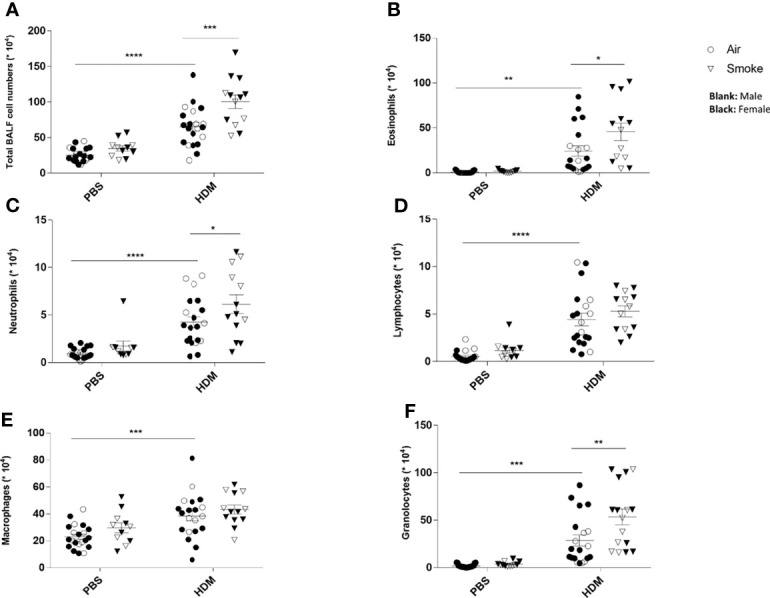
Prenatal and postnatal CS exposure induced additional inflammatory cell infiltration into the lungs of offspring upon HDM challenge. Prenatally and postnatally air- and CS-exposed offspring were intranasally (i.n.) sensitized with PBS or house dust mite (HDM) on day 37 and challenged on days 43-47 intranasally with PBS or HDM and sacrificed one day after last HDM/PBS challenge. Lungs were lavaged and BALF was collected for total **(A)** and differential BAL cell counts, including eosinophils **(B)**, neutrophils **(C)**, lymphocytes **(D)**, macrophages **(E)** and granulocytes **(F)**. *P < 0.05, **P < 0.01, ***P < 0.001 and ****P < 0.0001; as analyzed with two-way ANOVA followed by Bonferroni’s multiple comparisons test.

To investigate the extent of pulmonary inflammation and inflammatory cell infiltration into the airways, BALF was analyzed in PBS- and HDM-treated offspring born to air- and smoke-exposed mothers (**Figure 3**). First of all, no significant differences were observed in the number of eosinophils, neutrophils, lymphocytes and macrophages in PBS-treated offspring from air- and CS-exposed offspring (**Figures 3A–F**). The total number of inflammatory cells (**Figure 3A**), eosinophils (**Figure 3B**), neutrophils (**Figure 3C**) and lymphocytes (**Figure 3D**) was significantly higher in the BALF of HDM-treated offspring pre- and postnatally air exposed to air compared to PBS-treated offspring. This is in line with previous mouse models of HDM-induced allergic asthma (40, 41). Prenatal and postnatal CS exposure caused an increase in the number of total BALF cell counts (**Figure 3A**), eosinophils (**Figure 3B**) and neutrophils (**Figure 3C**) in the airways of HDM-treated offspring. However, the number of lymphocytes and macrophages did not significantly differ between HDM-treated offspring born to air-exposed mothers compared to offspring from CS-exposed mothers.

### Prenatal and Postnatal CS Exposure Resulted in Changes in T Cell Subsets in the Lungs of Offspring Upon HDM Challenge

**Figure 4:**
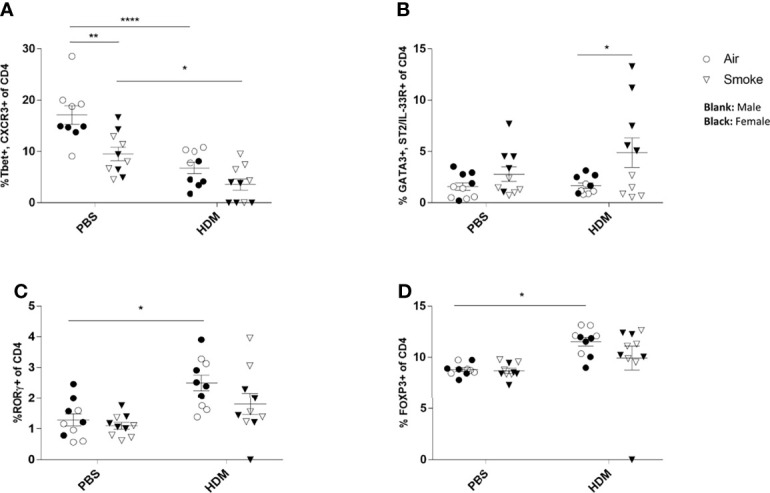
Prenatal and postnatal CS exposure resulted in changes in T cell subsets in the lungs of offspring upon HDM challenge. Prenatally and postnatally air- and CS-exposed offspring were intranasally (i.n.) sensitized with PBS or house dust mite (HDM) on day 37 and challenged on days 43-47 intranasally with PBS or HDM and sacrificed one day after last HDM/PBS challenge. **(A)** The percentage of Th1 cells (Tbet+, CXCR3+ of CD4+ cells), **(B)** Th2 cells (GATA3+, ST2/IL-33R+ of CD4+ cells), **(C)** Th17 cells (RORγ+ of CD4+ cells) and **(D)** regulatory T cells (Tregs) (FoxP3+ of CD4+ cells) were analyzed in lung cell suspensions. Data are presented as mean ± SEM. *P < 0.05, **P < 0.01 and ****P < 0.0001; as analyzed with two-way ANOVA followed by Bonferroni’s multiple comparisons test.

To investigate the pulmonary inflammatory response more in-depth, lung cell suspensions were analyzed for T cell subsets *via* FACS analysis (**Figure 4**). Prenatal and postnatal CS exposure significantly reduced the frequency of Th1 cells (measured as CD4+ Tbet+ CXCR3+ cells) in the lungs of PBS-treated offspring compared to offspring born to air-exposed mothers (**Figure 4A**). Although the HDM challenge in the air-exposed offspring significantly reduced the Th1 cells in the lungs, HDM-treated offspring from CS-exposed mothers did not demonstrate any additional effect on the Th1 cell population in the lungs (**Figure 4A**).

Moreover, our data revealed that the Th2 (measured as CD4+GATA3+ST2/IL-33R+ cells) cell population increased significantly after the HDM challenge in the airways of offspring from CS-exposed mothers compared to offspring born to air-exposed mothers (**Figure 4B**). However, no significant changes were observed between PBS and HDM-treated offspring from air-exposed mothers as well as from CS-exposed mothers (**Figure 4B**). We further analyzed the lung homogenates for the Treg and Th17 cell frequency (**Figures 4C, D**). The frequency of the CD4+RORγt+ Th17 cells increased significantly in the HDM-treated offspring prenatally and postnatally exposed to air compared to PBS-treated mice (**Figure 4C**). Similar results were obtained for CD4+Foxp3+ T-reg cells (**Figure 4D**). Prenatal and postnatal CS exposure did not further increase the CD4+RORγt+ Th17 and CD4+Foxp3+ T-reg cell frequency (**Figures 4C, D**).

### Prenatal and Postnatal CS Exposure Resulted in Additional Cytokine Secretion in the Lungs of Offspring Upon HDM Challenge

To determine the effect of the HDM challenge on lung cytokine release, the concentration of the Th2-associated cytokines IL-4, IL-5 and IL-13 was determined in the BALF of offspring from air-and CS-exposed mothers.

**Figure 5:**
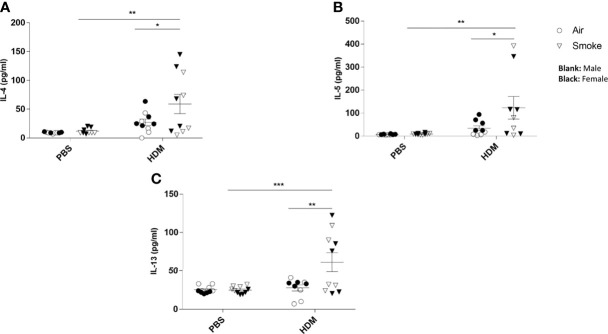
Prenatal and postnatal CS exposure resulted in additional cytokine secretion in the lungs of offspring upon HDM challenge. Prenatally and postnatally air- and CS-exposed offspring were intranasally (i.n.) sensitized with PBS or house dust mite (HDM) on day 37 and challenged on days 43-47 intranasally with PBS or HDM and sacrificed one day after last HDM/PBS challenge. **(A)** IL-4, **(B)** IL-5 and **(C)** IL-13 concentrations were measured in BALF. Data are presented as mean ± SEM. *P < 0.05, **P < 0.01 and ***P < 0.001; as analyzed with two-way ANOVA followed by Bonferroni’s multiple comparisons test.

No difference in cytokine release could be measured in BALF isolated from the offspring of CS- or air-exposed mothers when challenged with PBS (**Figure 5**). When the offspring were challenged with HDM, significant increased concentrations of IL-4 (**Figure 5A**), IL-5 (**Figure 5B**) and IL-13 (**Figure 5C**) were observed in offspring from CS-exposed mothers compared to air-exposed mothers. In addition, a HDM challenge in animals born to CS-exposed mothers resulted in significant increased levels of IL-4 (**Figure 5A**), IL-5 (**Figure 5B**) and IL-13 (**Figure 5C**) when compared to a PBS challenge. This effect of HDM was not observed in BALF from offspring of air-exposed mothers (**Figures 5A–C**).

### Prenatal and Postnatal CS Exposure Increased the IL-4, IL-5 and IL-13 Production After *Ex Vivo* Restimulation of Lung Cells From Offspring Upon HDM Challenge

**Figure 6:**
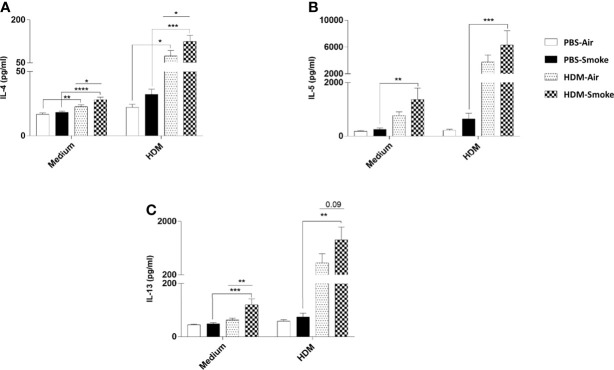
Prenatal and postnatal CS exposure increased the IL-4, IL-5 and IL-13 production after ex-vivo restimulation of lung cells from offspring upon HDM challenge. Prenatally and postnatally air- and CS-exposed offspring were intranasally (i.n.) sensitized with PBS or house dust mite (HDM) on day 37 and challenged on days 43-47 intranasally with PBS or HDM and sacrificed one day after last HDM/PBS challenge. Lung cell suspensions from the offspring were *ex vivo* restimulated with medium or HDM for 4 days (37°C, 5% CO2). **(A)** IL-4, **(B)** IL-5 and **(C)** IL-13 concentrations were measured in the supernatants (picogram per milliliter). Data are presented as mean ± SEM, n = 7–9 mice/group. Data are presented as mean ± SEM. *P < 0.05, **P < 0.01, ***P < 0.001 and ****P < 0.0001; as analyzed with two-way ANOVA followed by Bonferroni’s multiple comparisons test (N=10).

Subsequently, the cytokine release in offspring lung homogenates unchallenged (medium) or challenged with HDM was investigated. There was no difference between IL-4, IL-5 nor IL-13 cytokine release in the unchallenged lung homogenates from PBS-treated offspring of air- and CS-exposed mothers upon PBS treatment (**Figure 6**). However, an increase in basal levels of IL-4 and IL-13 could be detected in the unchallenged lung homogenates from HDM-treated offspring born to CS-exposed mothers compared to air-exposed mothers. When the offspring lung homogenates were challenged with HDM, no difference in cytokine release could be detected between PBS-treated air- and PBS-treated CS-exposed mothers (**Figure 6**). In contrast, challenged lung homogenates from HDM-treated offspring born to CS-exposed mothers showed increased levels of IL-4 (**Figure 6A**) and IL-13 (p<0.09) (**Figure 6C**) release compared to PBS-treated air-exposed mothers.

### Prenatal and Postnatal CS Exposure Increased the HDM-Specific IgE and IgG1 Release in Serum From Offspring Upon HDM Challenge

**Figure 7:**
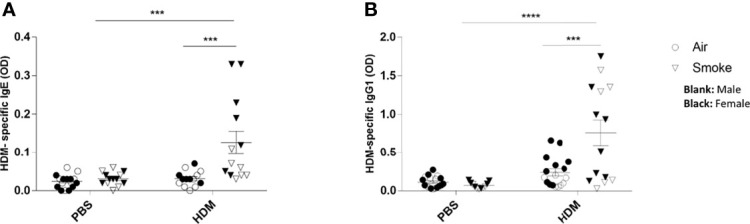
Prenatal and postnatal CS exposure followed increased the HDM-specific IgE and IgG1 release in serum from offspring upon HDM challenge. Prenatally and postnatally air- and CS-exposed offspring were intranasally (i.n.) sensitized with PBS or house dust mite (HDM) on day 37 and challenged on days 43-47 intranasally with PBS or HDM and sacrificed one day after last HDM/PBS challenge. HDM-IgE **(A)** and HDM-IgG1 **(B)** were measured in serum by means of ELISA. Results are shown as mean ± SEM. Statistical significance of differences was tested using a One-Way ANOVA with *post hoc* Bonferroni’s multiple comparisons test. ***P < 0.001 and ****P < 0.0001; as analyzed with two-way ANOVA followed by Bonferroni’s multiple comparisons test.

Finally, the adverse effects of prenatal and postnatal CS exposure on the serum HDM-specific IgE and HDM-specific IgG1 release were assessed (**Figures 7A, B**). Serum immunoglobulins levels of HDM-specific IgE were significantly increased upon a HDM challenge in offspring born to CS-exposed mothers compared to offspring from air-exposed mothers (**Figure 7A**). Similar to HDM-specific IgE serum concentrations, elevated serum levels of HDM-specific IgG1 upon the HDM challenge in offspring from CS-exposed mothers compared to offspring from air-exposed mothers were observed (**Figure 7B**). No significant changes were observed between HDM-treated offspring born to air-exposed mothers compared to PBS-treated offspring born to air-exposed mothers. Moreover, no changes were detected in HDM-specific IgE and HDM specific IgG1 serum levels between offspring from CS- and air-exposed mothers after PBS treatment. Effect of HDM is only observed in offspring born to CS-exposed mothers (**Figures 7A, B**).

## Discussion

Increased exposure to household air pollution (HAP) and ambient air pollution (AAP) has become one of the world’s major environmental health threats. Substantial evidence indicates that HAP and AAP is associated with enhanced prevalence of different types of pollution-related diseases, including allergic asthma (32, 42–45). According to the Environmental Protection Agency, the air quality of the homes and offices can be up to five times (and in some cases up to 100 times) more polluted than the air outside (46). In developing and developed countries, environmental cigarette smoke exposure (ETS) is one of the main sources of indoor pollution (HAP) (16). Exposure of nonsmoking pregnant women to household air pollution is linked to a variety of adverse perinatal and postnatal outcomes including lower birthweight, smaller head circumference and stillbirth (20). Moreover, results from different epidemiological and experimental studies indicate that there is a strong association between maternal HAP, specifically CS exposure, and the development of allergic diseases that often persists into later life (47–50). Children born to smoking mothers tend to have increased risk of impaired lung function, wheeze and asthma (51). In the current study, we aimed to provide a better understanding of the events involved in the development or exacerbation of allergic airway responses in the offspring parentally and postnatally exposed to CS. Therefore, mothers were daily exposed to CS during pregnancy and lactation and the allergic immune response in the offspring were mimicked by using a HDM-induced allergic asthma model.

HDM-induced allergic asthma models are recognized as relevant murine models representing similar pathophysiological symptoms as observed in human asthma, including airway resistance, increased airway inflammation and Th2 polarization (35, 52, 53). In line with previous studies, we found that 5 days exposure to HDM lead to enhanced airway resistance along with elevated total number of inflammatory cells, eosinophils, lymphocytes and neutrophils and cytokines in the BALF of HDM-sensitized offspring (41, 54).

The current study provides insight into the effect of an allergen, HDM, in offspring prenatally and postnatally exposed to CS with respect to immune cell recruitment into the lung. HDM treatment of offspring prenatally and postnatally exposed to air led to significant increases in total BALF cell counts, macrophages, eosinophil, lymphocytes and neutrophils compared to PBS-treated offspring born to air-exposed mothers, which is in line with previous studies (55, 56). Ferrini et al.,observed that HDM treatment in offspring prenatally exposed to air causes increased eosinophil numbers, IL-4, IL-5 and IL-13 release in BALF samples (55). In this study, HDM-treated offspring born to mothers prenatally and postnatally exposed to CS exhibited significantly higher eosinophil and neutrophil counts in the lungs accompanied with increased Th2 cytokines in BALF compared to HDM-treated offspring from air-exposed mothers. Christensen et al., confirmed that prenatal CS exposure followed by HDM challenge induced a pronounced exacerbation in the level of eosinophilic inflammation (56). Moreover, another study indicated that prenatal exposure to CS elevated the HDM-induced numbers of neutrophils (55).

To further investigate the immunological consequences of prenatal and postnatal CS exposure on HDM-induced allergic immune responses, the T cell subsets in the lungs, Th2 cytokine release in BALF, as well as the *ex vivo* immune response of lung cells re-stimulated with HDM, were investigated. Th1 cell subsets in the lungs were markedly lower after PBS treatment in offspring from CS-exposed mothers compared to offspring from air-exposed mothers. On the other hand, prenatal and postnatal CS exposure, caused an increased Th2 cell frequency in the offspring lungs upon HDM treatment compared to offspring from air-exposed mothers. Dysregulation of the Th1/Th2 cell balance, measured as a shift towards an increased Th2 cell-mediated immune response against allergens, plays an important role in allergic asthma development (57–59). The underlying mechanism on how prenatal and postnatal CS exposure leads to more Th2 cell frequencies is not clear yet. Several factors are involved in the increased offspring susceptibility, including a lower immunologic competence, a decreased capacity to detoxify carcinogens and higher cell proliferation rate (60, 61). It has been demonstrated that natural killer cells play an important role in alleviating airway allergic inflammatory responses. In a mouse model of HDM-induced airway inflammation, enhanced airway responses in offspring parentally exposed to CS were observed, which were associated with a reduction in CD3^−^CD19^−^NK1.1^+^CD94^+^ NK cell numbers (55). Moreover, epigenetic regulations have also been indicated as a plausible mechanistic explanation in prenatal CS exposure and exacerbated allergic responses (56). Prenatal CS and/or HDM exposure may lead to global DNA hypermethylation in lung, spleen and blood after HDM treatment and this effect was exacerbated in HDM-treated offspring prenatally exposed to CS (56). CS is an extremely complex and dynamic mixture containing more than 4000 harmful chemical compounds, including gases, nicotine and PM (62). Research shows that several CS constituents can cross the placenta barrier and affect fetal immune system development. For instance, polycyclic aromatic hydrocarbons (PAHs), as a widespread air pollutant also present in CS, can cross the placental barrier and prenatal and postnatal exposure to PAHs can result in developmental toxicity (60, 63). Other studies demonstrated that the toxins present in the gas phase of CS are also able to cross the placenta barrier leading to severe damage to the fetus (64, 65). Carbon monoxide, as a major combustible product of CS and in-door cooking, bypasses the placenta barrier and binds with fetal hemoglobin. This binding leads to carboxyhemoglobin (COHb) formation and fetal hypoxia (66). Moreover, nicotine can also be easily transferred across the placenta, which consequently leads to fetal toxicity and altered fetal development (67, 68). Interestingly, studies have indicated that cotinine levels in neonates exposed prenatally to CS are similar to that in active smokers, indicating active transfer of nicotine/cotinine from mothers to the fetus (69, 70). Nicotine has been shown to alter a wide range of immunological responses. For instance, nicotine treatment significantly increases Th2 differentiation in peripheral blood mononuclear cells (PBMCs) isolated from rheumatoid arthritis patients, as observed by an increase in IL-4 production and enhanced GATA3 expression (71).

In the current study, we demonstrated that HDM treatment in offspring prenatally and postnatally exposed to air, significantly increased the Th17 cell frequency in the lung. This is in line with enhanced Th17 cell subsets in the lungs observed in other murine HDM-induced asthma models (40). However, no additional effect of prenatal and postnatal CS exposure was observed on the Th17 cell frequency in the lung. Although there is an increasing interest in the role of Th17 responses in asthma pathology, it is currently unclear how Th2 and Th17 responses interact and drive the asthmatic phenotype. Analyzing Th2 and Th17 gene signatures in human asthmatic airways, it was found that they are inversely correlated suggesting a mutual exclusive relationship (72). This finding was further supported by the observations from a mouse model of HDM-induced asthma. Blocking IL-13 and IL-4, hallmark Th2 cytokines, increased IL-17 driven lung pathology following HDM exposure (72). Moreover, both human and mouse Th17 cells express the IL-13 receptor and exposure to IL-13 attenuates the production of IL-17 (73, 74). In the current study, offspring born to CS-exposed mothers showed increased numbers of Th2 cells and levels of Th2 cytokines in the lung following HDM exposure. This was not observed in the offspring of air-exposed mothers. Therefore, the increased allergic phenotype observed in the lungs of offspring from CS-exposed mothers might explain why there was no increase in lung Th17 cells in the offspring of CS-exposed mothers.

Another toxic compound present in the CS is PM. Smoking one cigarette exposes the human respiratory tract to between 15,000 and 40,000 μg of PM (75). CS particles are sized between 0.1 um to 1 um with a peak between 0.2 µm and 0.25 µm (also known as ultrafine particles) (76). *In vivo* and *in vitro* studies clearly indicate that particles of that size can be transferred across the placenta barrier and reach the fetus (77, 78). Using an *ex vivo* placenta perfusion model, Vidmar et al., demonstrated that silver nanoparticles are able to cross the placental barrier and could be detected in the fetal circulation in low but not negligible amounts (77). Like nicotine, PM has adverse effects on the immune cell responses. Mice exposed to PM_2.5_ showed an increased Th2 cell frequency in the lung as observed by overexpression of GATA3 and higher IL-4, IL-5 and IL-13 release in the BALF (78). In the current study, HDM-treated offspring born to CS-exposed mothers exhibit higher production of IL-4 in the BALF, which is in line with an increase in Th2 subsets observed in the lungs of these animals. IL-4 functions as autocrine growth factor and differentiation factor and exposure to IL-4 results in the proliferation and differentiation of naive T cells into effector cells (79). For instance, incubation of primary naïve human T-cells with IL-4 results in Th2-type lymphocyte clones, while incubation with anti-IL-4 prevents this differentiation (80).

Differentiated and activated Th2 cells produce the cytokines IL-4, IL-5 and IL-13. In the current study, we provide evidence that HDM-treated offspring born to CS-exposed mothers exhibit higher production of IL-4, IL-5 and IL-13 in BALF, which is in agreement with the increased presence of cells belonging to the Th2 subsets observed in the lungs of these animals. In addition to our observations in the BALF of the offspring, similar supporting evidence was found upon restimulation of the lung homogenates of the offspring. Offspring born to CS-exposed mothers showed increased basal levels of Th2-associated cytokines IL-4 and IL-13 compared to offspring born to air-exposed mothers. This was even further enhanced when the homogenates were activated with HDM, indicating that the HDM-treated offspring reacted to a HDM challenge with an increased Th2-associated allergic response. These cytokine responses were most pronounced in offspring treated with HDM born to CS-exposed mothers and compared to offspring from air-exposed mothers suggesting that prenatal CS exposure exacerbates HDM-induced allergic (or Th2-associated) cytokine responses in the offspring.

In addition to cellular responses, humoral responses, are also playing a critical role in allergic asthma development and dominated by IgE, IgG1 and IgG2 antibody responses. Here, we demonstrate that serum immunoglobulin levels of HDM-specific IgE and IgG1 were significantly higher upon HDM treatment in offspring born to CS-exposed mothers compared to offspring from air-exposed mothers. The underlying mechanism by which prenatal and postnatal exposure leads to higher IgE and IgG1 release is unclear. However, studies have demonstrated a strong link between IL-4 and IL-13 released by activated Th2 cells, and B cell class switching from IgM/D to IgG1 and IgE production (81, 82). For instance, B cells were unable to produce IgG and IgE antibodies in IL-13/IL-4 deficient mice (82). Moreover, studies have indicated that IL-13 also plays an important role in B cell class switching. Punnonen et al., showed that co-culture of human immature B cells derived from fetal bone marrow with activated CD4+ T cells in the presence of IL-13 leads to a significant increase in IgG and IgE synthesis (83).

Several lines of evidence have demonstrated increased lower respiratory illnesses and reduced pulmonary function in children born to smoking mothers (84–88). In the current study, HDM treatment in prenatally air-exposed offspring leads to an increased lung resistance, which is in line with previous findings (40). However, we did not detect any additional effect of HDM-treatment on the offspring born to CS-exposed mothers. Elevated lung resistance in HDM-treated offspring born to CS-exposed mothers has been reported, however in this study, mice were first sensitized with 100 µg HDM and further challenged with 50 µg HDM (two times over a period of two weeks), while in our study, mice were sensitized with 1 µg HDM and challenged with 10 µg HDM (5 times during one week) (55). These differences in HDM dosages used for sensitization and challenge might offer an explanation in the observed discrepancy between the effects on lung resistance. To further investigate the effect of prenatal and postnatal CS exposure on lung function of the offspring, dynamic lung compliance (Cdyn) and transpulmonary pressure (dPpl) was measured. Lung compliance refers to the ability of both lungs to stretch and expand during breathing (38, 89). Therefore, compliance is measured as the elastic recoil of the lungs, where low compliance indicates a stiff lung (high elastic recoil), and high compliance indicates a pliable lung (low elastic recoil). In practice, lung compliance can be divided into two different parameters, dynamic and static lung compliance (90). Static compliance refers to pulmonary compliance during periods when there is no airflow and muscles are relaxed such as during an inspiratory pause. Dynamic compliance describes the pulmonary compliance continuously measured during the rhythmic breathing and it has been defined as an indicator which shows both elastic recoil and airway resistance (91). Therefore, any changes in both lung tissue elasticity and surface elastic force might lead to changes in dynamic lung compliance and hence increase in pleural pressure. For instance, patients with emphysema have significant higher lung compliance due to the elastic tissue damage. Moreover, it is well known that lung compliance is inversely related to pleural pressure. Interestingly, our data, for the first time, revealed that prenatal and postnatal CS exposure induced a significant reduction in dynamic lung compliance (Cdyn) and a significant increase in pleural pressure (dPpl) in PBS-challenged mice. A possible explanation for the observed decrease in Cdyn might be due to a reduced surface tension. Surface tension has been indicated as one of the main factors affecting lung compliance. Alveolar type two cells are known to produce pulmonary surfactant which plays a crucial role in reducing surface tension necessary for a normal pulmonary compliance (92). However, altered surfactant release has been shown to play an important role in reduced lung compliance. Interestingly, in rat pups prenatally exposed to CS, a reduced dynamic compliance was measured (93), while decreased surfactant protein A levels in BALF were observed in ewes prenatally exposed to nicotine (94). The underlying mechanism by which prenatal and postnatal CS exposure affects children’s lung health is not defined yet. Among the toxic components found in CS, nicotine has gained a lot of attention as it poses several health hazards, as there is more and more evidence indicating that nicotine can easily cross the placental barrier (67, 68). According to the previous studies, the concentrations of nicotine measured in the umbilical vein are similar to the maternal vein nicotine (67, 88). Moreover, the level of nicotine in the fetal lung is similar to the blood level (88), indicating that the lungs of the offspring born to smoking mothers can be exposed to the same level of nicotine as seen in the blood of active smokers. Nicotine causes the production of oxygen radicals, while simultaneously lowering the lung’s antioxidant capacity (95). Therefore, nicotine might be one of the factors affecting the lung health in the neonates.

Another explanation might be found in the elevated levels of Th2-associated cytokines in the lungs, especially IL-13. Furthermore, excessive Th2 responses can lead to a robust fibroproliferative reactions in the lung, which consequently leads to less elasticity (96). We found that HDM treatment of offspring born to CS-exposed mothers leads to a significant increase in the IL-13 release into the BALF and *ex-vivo* restimulated lung tissue homogenates. Previous studies indicated that overexpression of IL-13, as a Th2 type cytokine, induces inflammation and airway remodeling (97, 98). Moreover, in an ex vivo model, IL-13 treatment in fibroblasts isolated from bronchial biopsies in patients with mild asthma and normal controls resulted in significant increase in collagen type-1 production, which consequently leads to reduced lung elasticity (99). Th2 type cytokines have also been linked to altered surfactant secretion in the lung, consequently leading to reduced dynamic lung compliance. For instance, IL-13 treatment significantly reduced mRNA and protein level of surfactant protein D (SP-D) in adult human alveolar type II cells *in vitro* (100). Previous studies have highlighted the role of sex differences in adaptive immune responses. For instance, women exhibit more activated CD4^+^ T cells and CD8^+^ T cells compared to men in an *in vitro* stimulation model of PBMCs with phytohaemagglutinin (101). Moreover, *in vitro* polyclonal activation of human PBMCs with mitogen phytohemagglutinin caused higher IL-4 and IL-10 cytokine production in female PMBCs than in male PBMCs (102). In the current study, our data also indicated gender-specific differences in HDM-induced immune responses in the offspring, where female offspring born to CS-exposed mothers exhibit higher immune responses than male offspring from CS-exposed mothers. This finding is in agreement with previous data of Fuseini et al., who found that intranasal administration of HDM to female BALB/c mice lead to higher BALF and lung IL-5, IL-13 and IL-17A protein expression compared to HDM-challenged male mice (103).

The current study has several limitations. Firstly, we used CS as a proxy for HAP. We recognize that this model mainly utilized mainstream CS exposure, while HAP might more revolve around sidestream CS exposure. Since CS is a component of HAP, CS might not fully encapsulate all the effects of HAP. However HAP, in contrast to CS, has not been fully characterized yet and it is currently impossible to obtain representative fully characterized and validated fractions for use in preclinical experiments. Therefore, we opted to utilize a well-known model based on the use of CS from reference cigarettes. When validated HAP fractions become available, future studies should compare HAP to CS to determine overlap and differences on offspring health with regard to allergic asthma. Secondly, in the current study, we exposed the dams during pregnancy and lactation starting at day 4 of gestation. In real life, exposure of aspiring mothers most likely already occurs before, and continues beyond, conception. Although we recognize the discrepancy, CS exposure before mating might have resulted in differences in mating results between air- and CS-exposed females, ultimately leading to insufficient litter for subsequent follow-up research. Despite these limitations, the current study has provided a biological plausibility for the epidemiological studies indicating that offspring born to smoking mothers and/or a born in an environment with high exposure to HAP are more likely to develop allergic diseases later in life. Although more in-depth studies are required to further investigate the maternal CS effect on offspring, our results clearly indicate that prenatal and postnatal CS exposure increases the susceptibility of offspring (particularly the females) to allergic immune responses.

## Data Availability Statement

The original contributions presented in the study are included in the article/**Supplementary Material**. Further inquiries can be directed to the corresponding author.

## Ethics Statement

The animal study was reviewed and approved by Dierexperimentencommissie Utrecht.

## Author Contributions

SB, HJ, JB, JG, and GF designed research. HJ, MD, TL-M, and IA performed research. HJ and TL analyzed data. HJ, GF, JB, and SB wrote the paper. All authors contributed to the article and approved the submitted version.

## Conflict of Interest

JG and JB are employees of Danone Nutricia Research.

The remaining authors declare that the research was conducted in the absence of any commercial or financial relationships that could be construed as a potential conflict of interest.

## Publisher’s Note

All claims expressed in this article are solely those of the authors and do not necessarily represent those of their affiliated organizations, or those of the publisher, the editors and the reviewers. Any product that may be evaluated in this article, or claim that may be made by its manufacturer, is not guaranteed or endorsed by the publisher.
