# Supplementary material for: Prenatal and Postnatal Cigarette Smoke Exposure Is Associated With Increased Risk of Exacerbated Allergic Airway Immune Responses: A Preclinical Mouse Model
Source: Front Immunol. 2021 Dec 23;12:797376. doi: 10.3389/fimmu.2021.797376 (PMC8732376; doi:10.3389/fimmu.2021.797376)
Supplement: Supplementary file 1 [file DataSheet_1.docx]

Supplementary Material

#
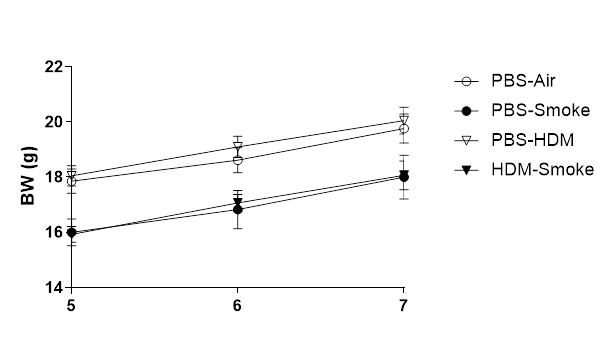
Supplementary Figures

Supplementary figure 1. Body weight measurements in offspring

Offspring were weighed weekly for 3 consecutive weeks (postnatal week 5, 6 and 7).
