# Supplementary figures and images for: Prenatal and Postnatal Cigarette Smoke Exposure Is Associated With Increased Risk of Exacerbated Allergic Airway Immune Responses: A Preclinical Mouse Model
Source: Front Immunol. 2021 Dec 23;12:797376. doi: 10.3389/fimmu.2021.797376 (PMC8732376; doi:10.3389/fimmu.2021.797376)

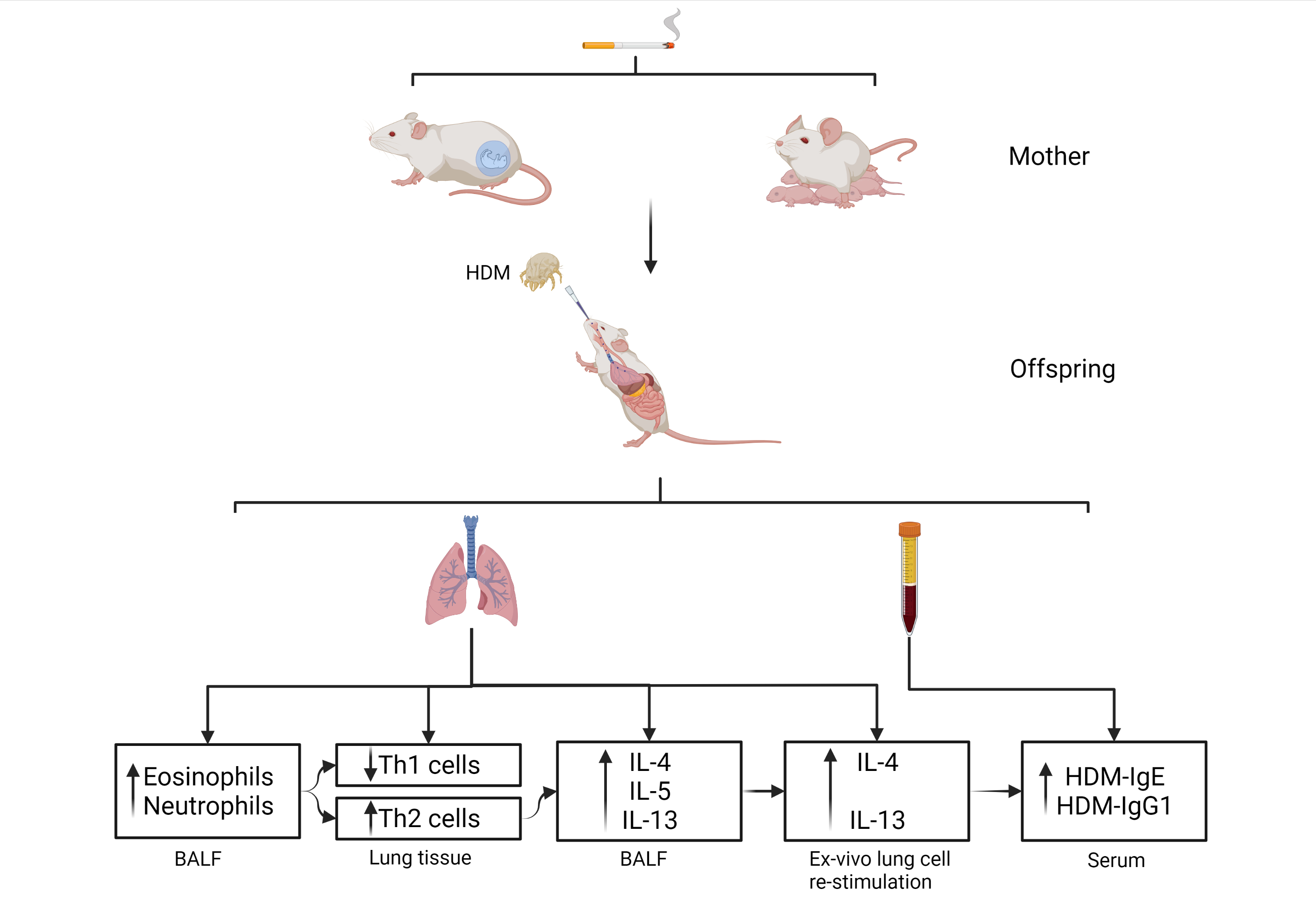

Supplement: Supplementary file 2 [file Image_1.jpeg]
